# Understanding natural genetic variation for grain phytic acid content and functional marker development for phytic acid-related genes in rice

**DOI:** 10.1186/s12870-022-03831-2

**Published:** 2022-09-17

**Authors:** Muhammed Azharudheen TP, Awadhesh Kumar, Chandrappa Anilkumar, Rameswar Prasad Sah, Sasmita Behera, Bishnu Charan Marndi

**Affiliations:** 1 Crop Improvement Division, ICAR-National Rice Research Institute, Cuttack, India; 2 Crop Physiology and Biochemistry Division, ICAR-National Rice Research Institute, Cuttack, India

**Keywords:** Gene-based marker, Phytic acid, Rice, *SPDT*

## Abstract

**Background:**

The nutritional value of rice can be improved by developing varieties with optimum levels of grain phytic acid (PA). Artificial low-PA mutants with impaired PA biosynthesis have been developed in rice through induced mutagenesis. However, low-PA mutant stocks with drastically reduced grain PA content have poor breeding potential, and their use in rice breeding is restricted due to their detrimental pleiotropic effects, which include decreased seed viability, low grain weight, and low seed yield. Therefore, it is necessary to take advantage of the natural variation in grain PA content in order to reduce the PA content to an ideal level without compromising the crop's agronomic performance. Natural genetic diversity in grain PA content has not been thoroughly examined among elite genetic stocks. Additionally, given grain PA content as a quantitative trait driven by polygenes, DNA marker-assisted selection may be required for manipulation of such a trait; however, informative DNA markers for PA content have not yet been identified in rice. Here we investigated and dissected natural genetic variation and genetic variability components for grain PA content in rice varieties cultivated in Eastern and North-Eastern India during the last 50 years. We developed novel gene-based markers for the low-PA-related candidate genes in rice germplasm, and their allelic diversity and association with natural variation in grain PA content were studied.

**Results:**

A wide (0.3-2.8%), significant variation for grain PA content, with decade-wise and ecology-wise differences, was observed among rice varieties. Significant genotype x environment interaction suggested polygenic inheritance. The novel candidate gene-based markers detected 43 alleles in the rice varieties. The new markers were found highly informative as indicated by PIC values (0.11–0.65; average: 0.34) and coverage of total diversity. Marker alleles developed from two putative transporter genes viz*., SPDT* and *OsPT8* were significantly associated with grain PA variation assayed on the panel. A 201 bp allele at the 3’ UTR of *SPDT* gene was negatively associated with grain PA content and explained 7.84% of the phenotypic variation. A rare allele in the coding sequence of *OsPT8* gene was positively associated with grain PA content which explained phenotypic variation of 18.49%.

**Conclusion:**

Natural variation in grain PA content is substantial and is mostly controlled by genetic factors. The unique DNA markers linked with PA content have significant potential as genomic resources for the development of low-PA rice varieties through genomics-assisted breeding procedures.

**Supplementary Information:**

The online version contains supplementary material available at 10.1186/s12870-022-03831-2.

## Background

Up to 85 percent of the phosphate (P) in rice grains and other plants is stored as phytic acid (PA) (myo-inositol-1,2,3,4,5,6-hexakisphosphate) [1]. PA chelates cations and limits digestive enzyme activity by binding to certain amino acid residues [2]. As a result, the bioavailability of minerals such as iron, zinc, potassium, calcium, and magnesium is reduced [3]. PA is destroyed by the phytase enzyme during digestion in ruminants. While monogastric animals (such as humans) have minimal phytase activity in their digestive tracts, this limits phytate breakdown in food to approximately 10 percent; over 90 percent is expelled, resulting in eutrophication of aquatic bodies [4]. In the world's least developed regions, including South Asian countries, rice is the most important staple food crop. Due to its low zinc and iron content, as well as its moderate to high levels of PA, rice is a poor source of vital micronutrients. Consequently, micronutrient malnutrition affects approximately 3 billion people worldwide who rely solely on rice to meet their calorie requirements [5, 6]. Rice grain quality is a broad term that encompasses a variety of grain appearance and compositional characteristics. The mineral content of grains is crucial, especially for rice-consuming communities with hidden hunger. To boost the bioavailability of Ca, Mg, Fe, and Zn in grain, the grain PA content must be decreased to an optimal level, thus developing low PA rice cultivars is a sustainable, cost-effective, and ecologically friendly approach of providing nutrient-rich rice to the world's rice consumers.

Through mutagenization, low-PA mutants have been developed that had much lower grain PA content [7–9]. When compared to wild-type seeds, these mutants had 45–95 percent less PA [10, 11]. SULTR-like phosphorus distribution transporter (*SPDT*) knockout mutants modify phosphorus distribution, resulting in a 30% drop in grain PA content. On the other hand, these induced mutations resulted in a 12.5–25.6 percent reduction in grain yield and a 7.8–26.3 percent reduction in seed viability. Due to the non-targeted mutagenesis used to generate these mutants, their agronomic performance was significantly inferior to that of conventionally bred cultivars [12, 13]. Such undesirable hereditary characteristics in mutant lines jeopardise their usefulness and prevent them from being selected in breeding programmes. Alternatively, genomic resources developed for the purpose of identifying naturally occurring genetic variants for grain PA are limited [14]. Recent study [2] reported a single nucleotide substitution in the *SPDT* gene that is associated with the low PA phenotype and increased mineral bioavailability in the rice variety Khira. Similarly, an increase of zinc bioavailability (up to 12.51 mg/kg) was reported in natural low-PA variants of rice lines [15].

The quantitative inheritance of PA content with influence by environmental variations [16] and difficulty in phenotyping hinders thorough dissection of its genetics. A better understanding on effects of genetic and environmental contribution to grain PA content along with genomic resources for selection of low PA genotypes will reward rice breeders. While, only two QTL for grain PA content were reported based on the natural variation, from the IR 64 × Azucena mapping population [17], few QTLs [18] and several candidate genes for low PA were identified from mutant genetic stocks (Supplementary Table S1). The candidate genes reported for low-PA in rice belongs to two categories: catalytic enzymes which are directly involved in PA biosynthesis and transporter genes associated with phosphorous homeostasis, transport and allocation [19].

Marker-assisted selection (MAS) using DNA markers greatly improve the efficiency of breeding, especially for a trait like grain PA content owing to the difficulty in phenotyping and polygenic inheritance. Availability of polymorphic and user-friendly DNA markers is a pre-requisite for a successful MAS programme. Linked random DNA markers are widely utilized in rice molecular breeding. Random markers are highly polymorphic, but their application in MAS is limited because such markers are only in partial LD with the targeted gene/trait. The genetic recombination between the random marker and the target gene reduces the accuracy of selection. Hence, genic/functional DNA markers, which are in complete LD with the targeted gene, needs to be developed from the polymorphic sites within the gene sequences governing the phenotypic variation for a particular trait. Genic markers never recombine with the targeted gene hence are highly predictive of the phenotype, and will facilitate efficient selection of favourable alleles in breeding programmes [20]. However, molecular marker resources developed for grain PA content in rice are limited. Identification of candidate genes for the trait and the mining of the alleles in natural populations are primarily required to develop the genic or functional markers [20, 21]. Candidate genes affecting grain PA content are reported in rice. But the allelic diversity studies in natural population and its correlation with grain PA content is yet to be explored. Hence, it would be useful to develop PCR-based genic markers for grain PA content improvement in MAS programmes.

Knowledge of the allelic diversity in low PA candidate genes and their effects would be helpful for genetic improvement towards reduction of PA content in rice. We [2] reported that a T-C mutation in the fifth exon of the *SPDT* gene causes spontaneous variation in grain PA content in rice as well as enhanced mineral nutrient bioavailability. However, a single SNP in the *SPDT* gene may not be sufficient to account for the entire diversity in grain PA level found in rice germplasm. The impact of polymorphism loci in other potential genes linked to grain PA content should be investigated. Hence, the current research focuses on i) examining natural variation for low-PA in a set of elite rice varieties developed over the last 50 years, (ii) developing novel candidate gene-based markers for low-PA related candidate genes in rice, and (iii) investigating the relationship between allelic diversity at low-PA related candidate genes and grain PA content variation. The information will aid in determining the role of genetic and non-genetic variation in rice PA content. In addition, novel candidate gene-based markers linked to grain PA content variation could be used in marker assisted development of low-PA rice varieties to ensure nutritional security.

## Results

### Phenotypic variation for grain PA content in rice germplasm

The PA content in rice grains recorded on two replications over two years was subjected to estimation of BLUP values, which were further used to estimate descriptive statistics (Table 1). The variation observed for grain PA content on the panel of 96 genotypes was highly significant (*p* ≤ 0.05). The PA content in grains ranged from 0.30% (Khira) to 2.98% (Manipuri black) with a mean of 1.45 percent (the grain PA content of 96 rice genotypes for the years 2020 and 2021 is given in Supplementary Fig. S1). Further, the third-degree statistic- skewness was found negligible, and the fourth-degree statistic- kurtosis was also found platykurtic.

**Table 1:** Descriptive statics of grain PA content in in the rice germplasm

**Mean**	**Phenotypic variance**	**Standard error**	**Range (%)**	**Skewness**	**Kurtosis**	**Shapiro-Wilks ‘*p*’**
1.45	0.14	0.04	0.30–2.98	0.68	2.98	0.03

**Fig. 1:**
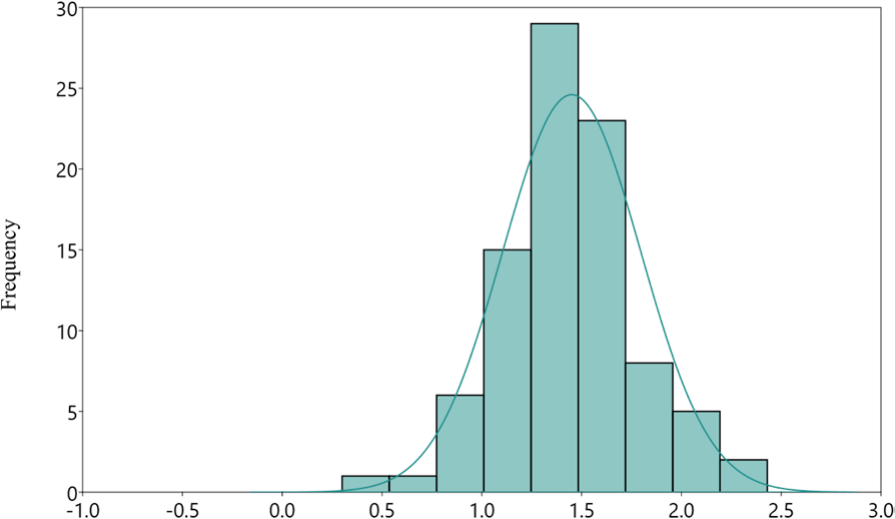
Distribution pattern of grain PA content measured in the association panel of 96 genotypes

The distribution pattern of phenotypic observations for grain PA content was depicted by plotting frequency distribution plots with overlaying normal curve (Fig. 1). The phenotypic observation of grain PA content was found normally distributed and confirmed by Shapiro–Wilk’s test [22].

The partition of total variation into variation due to genotype (G), year (Y) and G × Y interaction were found significant. Further, GCV, PCV, heritability in broad sense and genetic advance as percentage of mean (GA) were estimated with BLUP values over two years are presented in Table 2. Relatively moderate PCV (19.23%) and GCV (17.21%) exist for grain PA content and the difference between GCV and PCV was closer indicating a higher contribution of genetic make-up of genotypes to phenotypic expression. This is further evidenced by the high values of heritability, h^2^ (> 0.60) and genetic advance (> 20) for grain PA content. The moderate variance, high heritability and genetic advance for PA together help to understand the genetic nature of the trait and suggest possible selection for low-PA trait in the population.

**Table 2:** ANOVA and genetic estimates for mean grain PA content of rice genotypes evaluated over two years

**Genotypes (G)**	**Year (Y)**	**G × Y**	**GCV (%)**	**PCV (%)**	**h**^**2**^	**GA**
0.29**	0.70**	0.26**	17.21	19.23	0.81	20.33

*GCV* Genotypic coefficient of variance, *PCV* Phenotypic coefficient of variance, *h2* Heritability in broad sense, *GA* Genetic advance as percentage of mean

### Natural variation for grain PA content of rice varieties released in past 5 decades

**Fig. 2:**
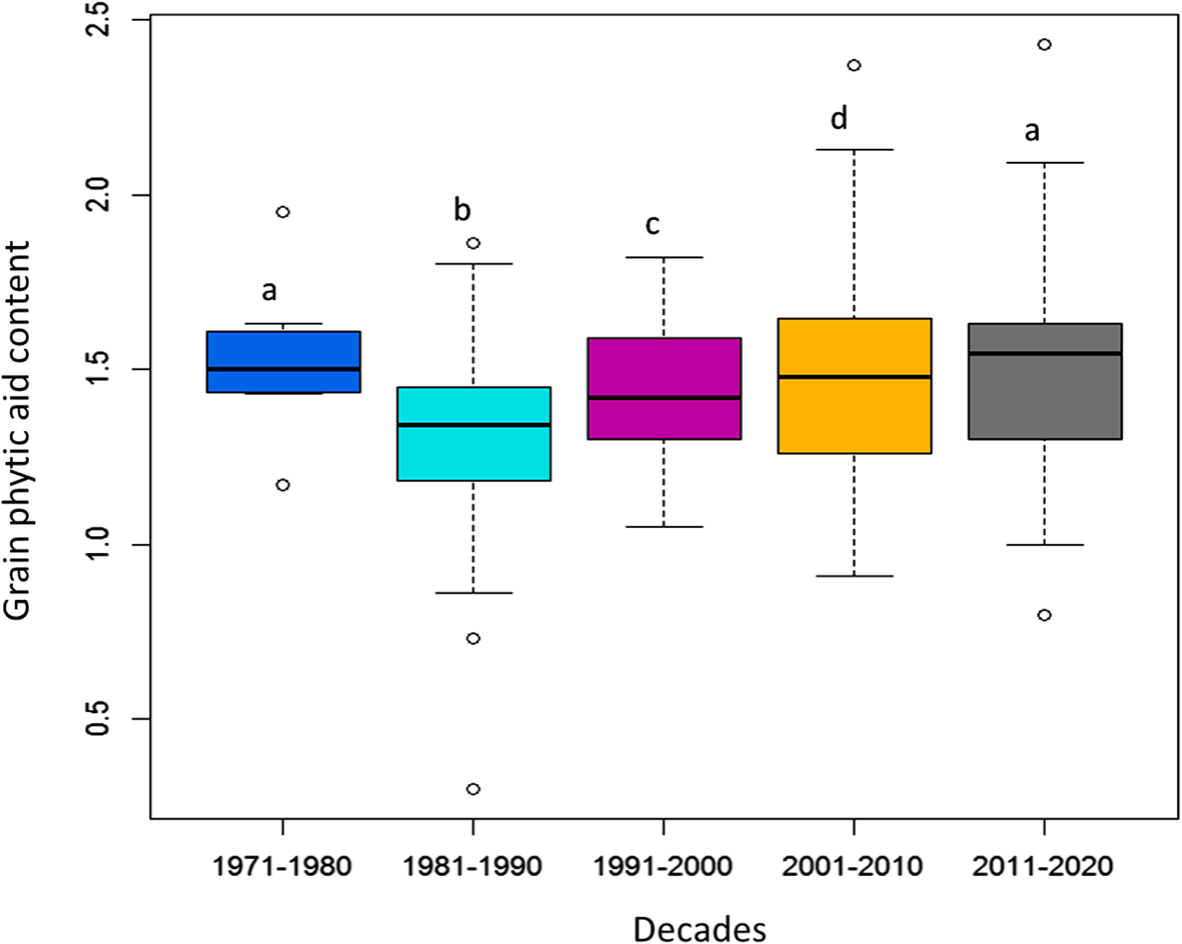
Grain PA variation in the 94 varieties released over the five decades (1971–2020). [The letters a, b, c, and d denote the significance of the mean PA content of rice varieties of between two decades. Different letters reflect statistical differences in mean PA content, with letters in common indicating that the differences are insignificant or marginally significant.]

The change in PA content in 94 varieties developed over 50 years (1971–2020) following the start of the green revolution was analysed by categorising the varieties into their corresponding release decades. The average PA content of cultivars developed between 1971 and 1980 was 1.53 percent, compared to 1.26 percent between 1981 and 1990, 1.44 percent between 1991 and 2000, 1.49 percent between 2001 and 2010, and 1.52 percent between 2011 and 2020. For the portrayal of temporal change in natural variation, the mean grain PA content of the rice varieties are depicted in a box-plot (Fig. 2). The change in PA content was statistically significant (DMRT significance) among cultivars developed between 1981 and 2010. Particularly, a considerable decline in PA content (1.26 percent) was seen between 1981 and 1990 compared to the other four decades. However, the PA content in 1971–1980 and 2011–2020 was not statistically significant and greater than in the previous decades (1981–2010). (Fig. 2).

### Comparison of grain PA variation in varieties developed for various growing ecologies

**Fig.3:**
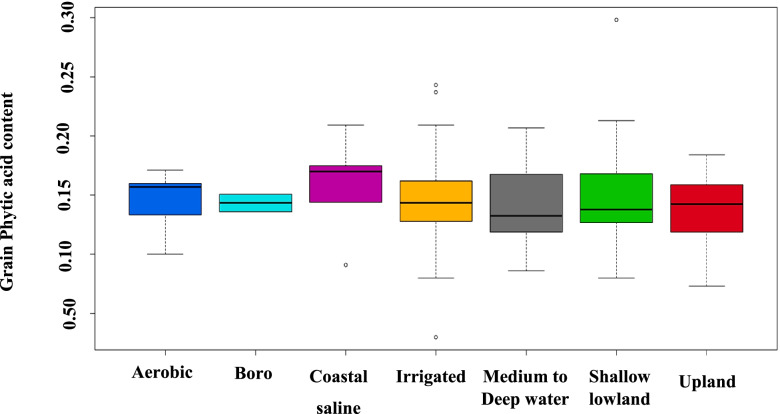
Variation of grain PA content in the 94 varieties released for different ecologies

The rice varieties were categorised on the basis of recommended ecologies for cultivation. The 94 varieties are categorised into 7 ecologies: Upland (16 varieties), Aerobic (7 varieties), Boro (2 varieties), Irrigated (32 varieties), Coastal saline (5 varieties), Shallow lowland (16 varieties), and Medium to Deep water (16 varieties). The average grain PA content of the rice varieties varied among the ecologies. Varieties developed for medium to deep water ecology included the least content of grain PA, followed by shallow lowland ecology. The varieties bred for coastal saline ecosystem had the highest grain PA content, followed by varieties released for the aerobic ecology. According to observations, the change from a habitat with a high-water requirement to one with a low water requirement is correlated with an increase in PA content. However, coastal saline ecology was an exception to this trend (Fig. 3).

### Polymorphic sites in LPA candidate genes and their allelic diversity in rice germplasm

Except for the gene *OsIPK1*, which catalyses the last step of PA biosynthesis in rice, polymorphic sites were curated from the nucleotide sequences of all the genes that have been reported as low-PA-related candidate genes (Table 3). Multiple polymorphic sites have been identified in the genes *OsLpa1* (4 sites), *OsMIK* (3 sites), *LPA* (3 sites), and *OsABCC13* (3 sites) (2 sites). A total of eighteen polymorphic sites were observed, of which 15 were SSRs and 3 were insertion/deletion (InDel) sites. The 5' UTR (5'- Untranslated region) of the gene contained 50% (9 sites) of the polymorphic sites, followed by the CDS (4 sites), intron (2 sites), 3' UTR (2 sites), and promoter sequence (1 site). On the basis of the polymorphic locations, fourteen genic markers were developed and the allelic diversity of 96 genotypes of rice germplasm was evaluated. All loci among the 96 lines exhibited genetic variation, with the exception of marker locus 11 (LOC Os03g04920, *OsMRP5/OsABCC13*) (Table 4). The panel revealed a total of 43 alleles, including 3 unique and 4 rare alleles. The average number of alleles per polymorphic amplified marker locus was 3. Among polymorphic markers, the number of alleles ranged from two (M1, M6, M7, M9) through three (M2, M3, M4, M5, and M14), four (M10 and M12), five (M13), and six (M8).

**Table 3:** Polymorphic sites observed and the markers developed for the PA-related candidate genes

**Gene**	**Locus ID**	**Marker locus**	**Polymorphic site**	**Primer sequence (5’-3’)**	**Annealing Temp (**^**0**^**C)**	**Expected amplicon (bp)**
*OsLpa1*	LOC_Os02g57400	M1	(AG)8 and (AGA)7 in 5' UTR	F: GGACACACACACAACTCCAC R: CTCTCGGCGTCCTCTTCTAC	58.99 59.34	213
*OsLpa1*	LOC_Os02g57400	M2	(T)12 and (G)10 in 5' UTR	F: GCTTCTCGTATCCAGCGTTG R: CCCGTCACCTTTCTTTGTCA	59.08 58.04	191
*OsLpa1*	LOC_Os02g57400	M3	(CTC)6 and (CGC)7 in 5' UTR	F: CAATGCGCCTCCTCAAAACC R: ACGGCAATGTAGAGGAGCTT	60.11 59.09	209
*OsLpa1*	LOC_Os02g57400	M4	(CT)10 in 5' UTR	F: CAACTCATGGGATGCAAGGC R: CGAAGCGGAAGAATCACGAG	59.54 59.08	203
*OsMIK*	LOC_Os03g52760	M5	(CTC)9 in 5' UTR	F: TCAACCGCCGCTTTTATTCC R: CATGGATGGAGGAGAAGGGG	59.19 59.23	157
*OsMIK*	LOC_Os03g52760	M6	(GGA)5 in CDS	F: CCCCACGCCTACTACTTCTT R: CTCGACCTCATCAAGCCCC	58.81 59.86	172
*OsMIK*	LOC_Os03g52760	M7	4 bp indel (AGGA) at 3' UTR	F: AATGAACTGACTTCGCTGCC R: GCACTCAGCGATTCCCATG	58.84 58.98	173
*LPA*	LOC_Os04g55800	M8	(ATCC)5 in intron	F: GATCGCGTCGCTGATAATGG R: GCCTGCATCCATCCATCAAG	59.29 59.04	244
*LPA*	LOC_Os04g55800	M9	(CAA)6 in 5' UTR	F: ATACACACCCCAAACCCTCC R: GCCGTTGTCATTGTAGCCAT	59.3 58.91	152
*LPA*	LOC_Os04g55800	M10	(CGC)6 in Intron	F: GCAATTGCGCATCCATAACG R: AGGAGTAGACGCGTGTGTAG	58.88 58.91	211
*OsIPK1*	LOC_Os04g56580	-	No SSR or InDel in promoter and gene sequence	-	-	-
*OsMRP5/* *OsABCC13*	LOC_Os03g04920	M11	(GAT)5 in CDS	F: TCTCCTTCGTGCTCTGTGTT R: CCATGACACCAACCAAGCAG	58.96 59.4	150
*OsMRP5/* *OsABCC13*	LOC_Os03g04920	M12	5 bp InDel in Promoter	F: ATAATATTCCGGCGCCTCGT R: GACCGATCCACAAGTCCACT	59.39 59.39	232
*SPDT/* *OsSultr3;4*	LOC_Os06g05160	M13	(AAG)18 in 3' UTR	F: AGAACAAGGTGCTTCTCGGA R: GAGATCAGCACCCGGAGTTA	58.95 58.89	222
*OsPT8*	LOC_Os10g30790	M14	(GAC)4 and a 3 bp InDel in CDS	F: GGGATCGGGGTGAGGAAC R: GTCATACACTACGCCGTCTG	59.09 58.18	194

**Table 4:** Allelic diversity parameters for the novel functional markers developed from PA-related genes in rice

**Marker locus**	**No. of alleles**	**Major allele frequency**	**Gene diversity**	**PIC**
M1	2	0.80	0.32	0.27
M2	3	0.54	0.60	0.53
M3	3	0.67	0.50	0.45
M4	3	0.61	0.52	0.44
M5	3	0.79	0.33	0.28
M6	2	0.92	0.15	0.14
M7	2	0.75	0.38	0.30
M8	6	0.43	0.64	0.57
M9	2	0.88	0.22	0.19
M10	4	0.35	0.71	0.65
M11	1	1.00	0.00	0.00
M12	4	0.88	0.23	0.22
M13	5	0.51	0.62	0.56
M14	3	0.94	0.12	0.11

**Fig. 4:**
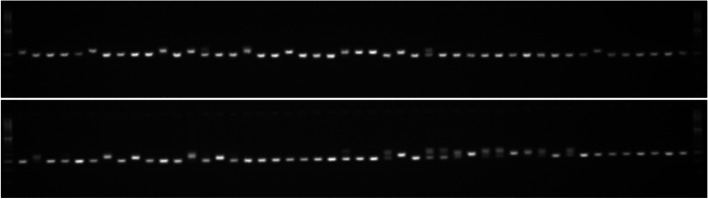
Representative image of allelic diversity at the (AAG)n genetic loci of 3’ UTR of the *SPDT* gene among 96 rice genotypes (uncropped full length gel image is provided in the Supplementary Information as Supplementary Fig. S2)

With six alleles, including one unique and one rare variant, the third intron of the *LPA* gene locus (ATCC)n was found to be a marker with a high degree of variability. The (AAG)n locus in the 3' UTR of the *SPDT* gene likewise exhibited a significant degree of genetic variability, with 5 alleles, one of which was a rare allele (Fig. 4). The gene diversity of the low-PA-related candidate genes assayed on the 96 genotypes ranged from 0.12 to 0.71, with an average of 0.38. In terms of polymorphism information content (PIC), the relative informativeness of the novel genic markers ranged from 0.11 (M14) to 0.65 (M10), with a mean PIC value of 0.34. Four markers, namely M2 (0.53), M8 (0.57), M10 (0.65), and M13 (0.56), had a PIC value greater than 0.5, rendering them extremely valuable for genetic dissection of PA genes, marker-trait association, and genomics-assisted breeding for low grain PA content.

**Fig. 5:**
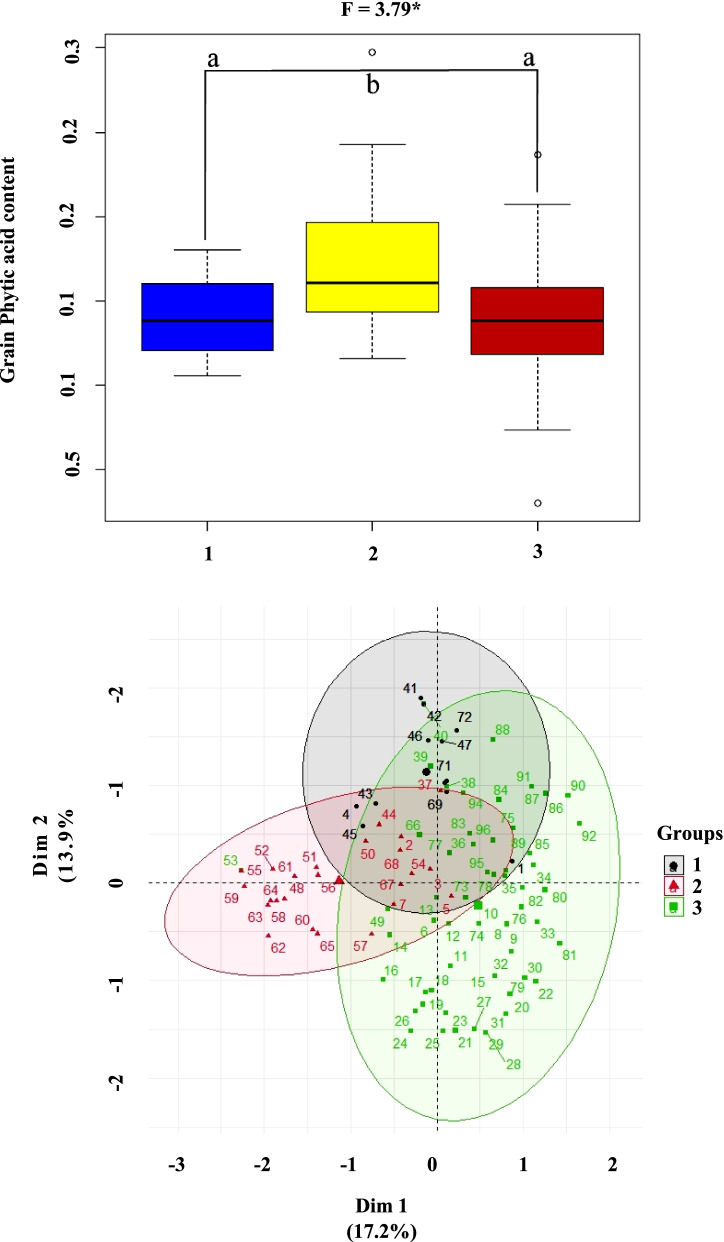
**A** Clustering of rice genotypes based on allelic diversity of new candidate gene markers developed for grain PA content in rice. **B** Whisker box-plot depicting mean grain PA content of clusters; F-value indicates significance among the clusters for the mean PA content; a, b indicates significance of means between any two clusters)

On the basis of the allelic diversity indicated by the genic markers, principal component analysis was performed, and the rice genotypes were grouped into three clusters using PCA (Fig. 5A). The allelic diversity-based construction of a dendrogram (rooted phylogenetic tree) yielded a similar grouping of genotypes (Supplementary Fig. S3). The three clusters are overlapping but differences in mean grain PA content was observed between clusters (Fig. 5B, Table 5).

**Table 5:** Difference in mean grain PA content among the clusters formed by allelic diversity of new functional markers

**Group**	**No. of genotypes**	**Mean PA content (%)**	**Std. deviation**
Cluster 1	12	1.40^a^	0.23
Cluster 2	24	1.71^b^	0.42
Cluster 3	60	1.36^a^	0.33
Whole population	96	1.45	

^a,^^b^ Indicates the significant differences in mean grain PA content between the clusters

The mean grain PA content in cluster 1 with its 12 genotypes was close to the population mean of 1.45%. In contrast, clusters 2 and 3 with 24 and 60 genotypes, respectively, had mean PA contents that were considerably higher (1.71%) and lower (1.36%) than the population mean, respectively. The mean grain PA content of cluster 2 was statistically significant compared to cluster 1 and cluster 3.

### Association between grain PA content and allelic variation at the low-PA gene loci

The Mixed Linear Model (MLM) method was used to identify association signals via the EMMA method in the GAPIT package of the R software. Out of the 18 polymorphic sites, two markers developed from the transporter genes *SPDT* and *OsPT8* were found to have genetic polymorphisms that were significantly correlated with the natural variation in the panel's grain PA content. In contrast, there was no correlation between grain PA content and allelic changes at polymorphic loci of genes directly involved in the PA biosynthesis pathway. Allelic variations at microsatellite loci (AAG)n in the 3' UTR of the *SPDT* gene on chromosome 6 were found to be strongly associated with grain PA content among rice genotypes. A 201 bp allele at this locus exhibited a significant negative correlation with grain PA content and accounted for 7.84% of the phenotypic variance observed in this study (Table 6). This suggests that marker-assisted selection for this gene contributes to the reduction of PA levels in breeding lines. The *OsPT8* allele, which has an amplicon size of 185 bp, was amplified specifically in rice genotypes with high levels of PA, and it was discovered that this association was highly significant. In the rice germplasm, this allele explained 18.49% of the overall phenotypic variation for grain PA content (Table 6).

**Table 6:** Association observed for grain PA content and allelic diversity of *SPDT* and *OsPT8* genes in a panel of 96 rice genotypes

**Gene**	**Chromosome**	**Marker loci**	**Observed Allele** **band size (bp)**	**R**^**2**^** (%)**
*SPDT*	6	M13	222	0.25
			231	0.16
			213	-
			201	7.84
			192	2.56
*OsPT8*	10	M14	194	1.44
			185	18.49
			203	0.81

For *SPDT* and *OsPT8* genes, we estimated the Tajima's D value, which was discovered to be 0.487 and 0.763, respectively. There were 95 haplotypes (h) at the *SPDT* gene and 115 at the *OsPT8* gene. Nucleotide diversity at the *SPDT* gene was 0.632, whereas it was 0.688 for the *OsPT8* gene (Supplementary Table S2).

## Discussion

Breeding efforts have been concentrated on increasing grain production potential since the 1960s, but improvements to grain quality characteristics only started in the decade 1981–1990 [23] and after. Only recently has low grain PA content been acknowledged as both a breeding target and a nutritional trait. PA is a naturally occurring, negatively charged chelating molecule that accounts for 65 to 85 percent of total seed phosphorus [24]. Phytate-mineral complex ions become insoluble, precipitate, and are not readily absorbed in non-ruminant animals. The scenario hinders intestinal absorption of minerals from food, even when high mineral content food is ingested with high phytate grains. As a result, mineral deficiency and hidden hunger are more prevalent in nations where rice is the predominant food source. Researchers from throughout the globe are concentrating their efforts on reducing grain PA levels without compromising grain production. For identification of superior donors and development of superior cultivars through breeding, it is necessary to: (i) understand natural genetic variation for grain PA content and effect of environment on accumulation of PA in grains, (ii) have varieties and natural variants with low-PA content for different rice ecologies, (iii) analyse the genetic nature of the low-PA trait, and (iv) have surrogate molecular markers available for deployment in genomic assisted breeding programmes [2].

### Natural variation for grain PA content in the rice germplasm

The PA content of rice is a double-edged sword, since either a decrease or a rise above a particular threshold is undesirable. The normal growth, development, and agronomic performance of the rice plant are impacted by a sharp drop in the PA content. The bioavailability of mineral micro-nutrients like zinc and iron is decreased as a result of PA, on the other hand. Therefore, if rice's nutritional value is to be increased, the PA content must be optimally minimised. In rice, induced mutagenesis and mutant genetic stocks have been used extensively to decipher the PA biosynthesis process and discover associated genes and QTLs. All of the candidate genes linked to the low-PA phenotype in rice were discovered in mutant populations. However, low-PA mutant stocks have low breeding value, and their use in rice breeding is restricted due to their detrimental pleiotropic effects, which include lower seed viability, low grain weight, and low seed yield [25]. It is conceivable to circumvent the need of mutant lines by discovering natural low PA genotypes for breeding, which would bar the limits imposed by mutants. There is natural variation for grain PA concentration in rice germplasm, however it has received the least attention in gene/QTL identification. Till now only two QTL for grain PA content have been identified based on the variability in natural population [26] that urges the need of thorough extensive research in the area. In order to identify 'evolutionarily conditioned' genotypes with optimal levels of PA content and superior agronomic features, it is crucial to harness the natural diversity for grain PA content in breeding programmes. In the present work, a panel of 96 rice genotypes, including two landraces and 94 modified cultivars bred for high yield, were phenotyped for two years for grain PA content. The germplasm exhibited substantial variation in grain PA content (0.3 percent to 2.98 percent).

First time in the global rice breeding history, natural variations for low grain PA were estimated in the varieties developed over decades, of which many are still under cultivation in larger area in the eastern India. PA content among these 94 varieties released in different decades (1971–2020) especially, during 1981–1990 was less (1.26%) compared to other decades (> 1.44%). This significant decrease in PA may be attributed to unintentional selection for low grain PA during varietal selections during 1981–1990. This information indicates availability of natural donors for low grain PA for future breeding programs. Apart from that, grain PA content found significantly associated with grain weight and varieties released during these decades were fine grain medium slender, that indirectly selected for low PA content [27]. Further, a specific pattern for natural variation in grain PA content in the rice varieties cultivated in different ecologies was observed, indicating possible influence of growing ecology in accumulating grain PA content. A decrease in PA content was recorded in ecologies with high water requirement (medium to deep water and shallow lowland ecologies) and gradual raise in PA content as water requirement reduces for cultivation i.e., PA in irrigated < boro < upland < aerobic varieties. However, the costal saline ecology were exceptions to this trend, which may be due to extreme abiotic stress (salinity and submergence stresses in coastal saline ecology). This difference in PA content in different ecologies may be attributing to genotypic efficiency for phosphorous acquisition, mobilization, translocation and PA biosynthesis [28, 29]. This result was supported by positive correlation between grain P content and grain PA content [30].

### Components of genetic variation for grain PA content in rice germplasm

PA, which has been reported to be a quantitatively inherited trait in rice, shows more variance across growing areas among cultivated rice varieties. To put it another way, genotype by environment interactions have a big impact on PA content in rice grains [16]. In the present study, the G × Y interaction was significant indicating the both genetic and non-genetic determinants influence grain PA content variation among genotypes of the panel. However, high heritability and closer difference between GCV and PCV evidence that genetic constitution of plants control the grain PA content with modest effect of G × Y interaction. This suggested that selection procedures in the appropriate growing environment may reward in identifying low PA genotypes. Genetic advance and heritability parameters also reiterated the genetic control of grain PA content. Genetic advance was relatively high indicating a good chance for genetic improvement by breeding; similar results for grain nutritive traits including PA were reported in earlier studies [16, 31]. Further, negligible skewness in the distribution of grain PA content indicates the additive gene interaction regulating the trait, while platykurtic distribution points involvement of several genes in controlling grain PA content [32, 33].

### Candidate gene-based markers for grain PA content

With respect to the mineral bioavailability and nutrition, reduced grain PA content is more crucial than increasing the micronutrient content of rice grain [34]. Difficulty in phenotyping and quantitative inheritance pattern of PA limited the development of genomic resources for practical utility. Identification of low grain PA genotypes in early segregating generations is tedious owing to the large population size to phenotype. Developments in biotechnology tools allow breeders to find contemporary ways to identify appropriate genotypes for difficult-to-phenotype traits [35]. Designing gene-specific polymorphic DNA markers for low PA allows more efficient selection in segregating populations. Candidate gene markers with high PIC value and co-dominance inheritance may specifically amplify target regions with less background noise. CAPS and InDel markers for differentiating lpa mutants from the wild type at the *OsLPA1* (LOC Os02g57400) locus were reported in a previous study [13]. Since those markers were specific to mutant regions, they did not find wide application in applied breeding. In our previous study, a non-synonymous SNP (T to C) in the fifth exon of *SPDT* gene was found responsible for the natural variation for grain PA content between rice varieties Khira and Phalguni [2]. However, due to lack of restriction site polymorphism, we could not develop an allele specific CAPS marker.

In the present study, eighteen polymorphic sites were found in the candidate genes associated with low PA content from which a set of 14 candidate gene-based markers were developed. The allelic diversity studies using gene-based markers identified 43 alleles at these loci upon assay on a panel of 96 rice genotypes. The average number of alleles per marker loci detected in the study (3.3) was much lower than what was previously reported with candidate gene-based SSR markers [36, 37]. This indicates the low PA-related candidate genes are highly conserved among 96 rice genotypes of the panel. However, the mean PIC value of 0.34 reported in the present study is higher than the previous reports for genic-markers [36, 37] and miRNA-SSR markers [38], specifically, four markers had PIC value of > 0.5. Nevertheless, higher PIC values for random SSR markers have been reported in previous studies [39–41]. Two markers, M13 and M14 (for genes *SPDT* and *OsPT8*) were found to be significantly associated with grain PA content. The marker M13 targeting the (AAG)n length polymorphism in 3’ un-translated region of the *SPDT* gene is especially important for marker-trait association for low PA and useful in marker aided selections since it meets all the specifications required for an ideal DNA marker. Though 5 alleles are observed in the 96 rice genotypes studied, many more alleles are expected to be present at this SSR loci in rice germplasm resources. Thus, it is possible to develop a high-yielding low PA rice line by harnessing natural variation for grain PA content by utilizing candidate gene-based marker resources.

### Association of allelic diversity of low-PA candidate genes with grain PA content

SSR polymorphisms in candidate gene sequences are reported to be associated with phenotypic expression of important agronomic traits in rice viz*.,* salinity tolerance [37], yield and nitrogen use efficiency [42]. In the present study, allelic diversity revealed by the genic-markers could classify the rice genotypes in to three clusters based on the genetic relatedness. However, a perfect clustering of genotypes based on the PA content was not observed, though most of the genotypes with high PA content (> 1.5%) was grouped in Cluster 2; most of the genotypes with low PA (< 1.0%) was grouped in Cluster 3 and genotypes with medium PA content (1–1.5%) were mainly present in Cluster 1. Inability to perfectly classify rice genotypes based on their PA content can be attributed to polygenic inheritance of the grain PA content in rice [43]. Whereas, marker allele-based assessment of allelic variation within the reported candidate genes captured only a portion of the diversity; there can be several unidentified genetic loci contributing to the natural variation for grain PA content in the 96 genotypes of the panel. Hence, it is imperative that the progress made in other cereal food crops like maize and barley for identifying genes/QTL governing grain PA content and in delineating the pathways affecting PA biosynthesis need to be emulated in rice [18, 44–47]. Further, the alleles of markers developed from *SPDT* and *OsPT8* genes were found significantly associated with natural variation for grain PA content. *SPDT* encodes a plasma-membrane localized transporter for phosphorus and functions as a switch to allocate phosphorus preferentially to the grains. The phosphate transporter gene *OsPT8* is crucial for the transfer of phosphorus between source and sink organs and between the embryo and endosperm of seeds. The number of haplotypes found for the *SPDT* gene (h = 95) in the rice germplasm was lower than that found for the *OsPT8* (h = 115). In the rice germplasm, it was also discovered that the nucleotide diversity (0.632 vs. 0.688) and Tajima D value (0.487 vs. 0.763) for the *SPDT* gene were lower than those for the *OsPT8* gene. The positive value for Tajima’s D for both the genes indicates lack of rare alleles for the gene locus in the cultivated rice germplasm and balancing selection and sudden population contraction. The markers for these two transporter genes have significant scope to deploy in marker aided breeding programs upon validation on large populations.

## Conclusions

To achieve nutritional security, it is critical to take into account and reduce major antinutritional factors such as PA that impair mineral bioavailability during food digestion. By breeding low grain PA cultivars, rice-dependent regions of the world may be able to ensure their nutritional security. Emphasis should be given to exploit the natural variation for grain PA content in breeding programmes. More genes/QTLs underlying grain PA content need to be identified from rice germplasm. Identification favourable alleles with cumulative effects on grain PA content and their DNA marker-assisted introgression in desired genetic backgrounds is the practically feasible strategy to manipulate grain PA content without adversely affecting the agronomic performance of the crop. The study reports novel candidate-gene based markers associated with grain PA content in rice. The low PA associated alleles of new markers have significant scope in identification of low PA genotypes, which can be best utilized in marker-aided breeding programs upon validation. These new candidate gene markers will enrich the low PA associated genomic resources in rice and play significant role in breeding programs aiming to develop low PA rice varieties.

## Materials and methods

### Genetic materials

Ninety-six rice genotypes, including 94 high yielding rice varieties bred and released for various ecologies of Eastern and North-Eastern India during past five decades and two popular landraces, one from Uttar Pradesh (Bindli) and another from Manipur state (Manipuri Black) constituted the genetic material for the study. Considering their extreme phenotypic levels for PA content, these two landraces were included in the study material. The varieties utilised in the study were released by the Government of India for cultivation in the designated ecologies, and the landraces were collected by the ICAR-National Rice Research Institute (ICAR-NRRI), Cuttack. The seeds of all the plant materials used in the study are available in the medium-term storage module of the National Rice Gene Bank, ICAR-NRRI, Cuttack. The authors obtained seeds from the National Rice Gene Bank, ICAR-NRRI, Cuttack. Any researcher may access seeds of plant materials used in the study for experimental purposes through Standard Material Transfer Agreement (SMTA) by submitting a request letter to the Director, ICAR-NRRI, Cuttack. This panel of 96 genotypes included rice varieties being cultivated in wide range of ecologies with different maturity durations, which were developed in due course of 50 years of rice varietal development programmes from 1970–2020. However, the low PA content was not the breeding objective while developing these varieties. Hence, the genetic material considered is chosen for the study was expected to show significant natural variation for PA content.

### Evaluation of experimental material

The pure seeds of experimental material were collected from nucleus seed of these rice varieties maintained at the ICAR- National Rice Research Institute, Cuttack, and seeds of landraces were collected from the gene bank, ICAR- National Rice Research Institute, Cuttack. Prior to experimentation the landraces were purified for two seasons through panicle progeny approach as per the standard protocol [48]. The genotypes of the panel were sown in raised nursery beds and 21 days-old seedlings were transplanted to main field. The field experimentation was conducted at experimental plots of ICAR-National Rice Research Institute, Cuttack, Odisha (20.4537° N, 85.9338° E) during wet seasons of 2020 and 2021. The genotypes were grown in randomised complete block design with 2 replications. The genotypes were grown in paired rows, each row with 30 plants and, row-to-row spacing of 20 cm and plant-to-plant spacing of 10 cm was maintained while transplanting. The experiment was conducted during wet seasons in both the years and package of practices recommended were followed to raise healthy crop. After complete maturity, grains were harvested from individual panicles of each genotype separately and shifted to laboratory for PA estimation.

### Phytic acid estimation

Using a moisture analyzer, the moisture content of dried harvested paddy was adjusted to 14 percent before processing. In a huller, the paddy was dehusked, then in a miller, it was milled (10%). Using an electric blender, the rice grains were powdered (100 mesh size). The PA was calculated using the fine rice powder obtained. The determination of P from PA after digestion with phytase and alkaline phosphatase enzymes was done using the Phytic acid test kit (Megazyme International Limited, Ireland) with certain modifications [49].

### Molecular marker assay

#### Primer design

The genomic sequences of PA-related candidate genes reported were retrieved from the whole rice genome from Rice Genome Annotation Project website [50]. The sequence was downloaded and the polymorphic sites in the gene sequences, Simple Sequence Repeats (SSRs) and insertion/deletion (InDels), were identified using Simple Sequence Repeat Identification Tool (SSRIT) [51] and NCBI Basic Local Alignment Search Tool (http://www.ncbi.nlm.nih.gov) [52]. The unique genomic sequences flanking the polymorphic sites were targeted for designing the primer pairs using the Primer3 software [53]. For genic regions having multiple adjacent SSR motifs, a single primer pair that includes all the adjacent SSRs in its amplifiable region was designed. While designing the primers, the parameters were selected such that the primer annealing temperature would be ~ 55 °C. For getting a specific, reproducible amplification in the PCR programme, the product size of the amplicons was selected in the range of 100–350 bp and with an optimum primer size of 20 bp. A set of 14 markers were designed from 6 reported low PA content related candidate genes in rice (Table 3).

### DNA extraction and PCR

The genomic DNA of the 96 rice genotypes was isolated from three-weeks old seedlings using CTAB method [54]. The genomic DNA and the candidate gene-based primers were diluted to get working concentrations of 20 ng/μl and 10 pico mole, respectively, for PCR amplification. PCR reactions were performed in a 10 µl final volume containing 20 ng of genomic DNA and the Taq Green Master Mix (Takara®). The reaction conditions were as follows: initial DNA denaturation at 94 °C for 4 min, followed by denaturation at 94 °C for 40 s, primer annealing at 55 °C for 40 s and elongation 72 °C for 1-min, was repeated for 40 cycles followed by final extension at 72 °C for 7 min, and final cooling at 4 °C. The amplified PCR products were subsequently resolved on 3.5% agarose gel in 0.5X TBE buffer, stained by ethidium bromide and visualized by UV trans-illuminator gel documentation system (Zenith Gel Pro CCD Gel Doc, Biozen Laboratories, India). The allelic variations at genic level for PA content was analysed using the novel markers designed from the candidate gene sequences.

### Statistical analysis

The PA was estimated in the grain for both the years and used for genetic and molecular analysis. Analysis of variance (ANOVA) was performed for testing the significance of PA content variance in grain over the years. The Fisher’s (F) statistic was used as test of significance of PA means. The average mean of the genotypes (G) was considered as fixed effect whereas, year (y), and the interaction of G × Y was considered to be random. Genetic parameters were also estimated to understand genetics of PA content variations among the test genotypes and to determine genetic and environmental effects regulating PA content in rice. These parameters include the genotypic coefficient of variance (GCV), phenotypic coefficient of variance (PCV), heritability (h^2^) and genetic advance as percentage of mean (GA) were calculated based on the published literature [55, 56]. Descriptive statistics, ANOVA, GCV, PCV, h^2^ and genetic advance for PA was performed using Indostat 7.5 software. The grain PA content variation among varieties released over different decades for different growing ecologies were compared using Duncan’s multiple range test (DMRT) to differentiate the significance in the means at *p* ≤ 0.05 significance level.

### Allelic diversity and Association analysis

Each amplified amplicon was considered as an allele and the amplicon sizes were calculated in comparison to 50 bp ladder marker (GeneDirex). An allele is considered unique when it is present in only one accession and alleles are designated as rare when they are present in less than 5% of the accessions. The relative value of each primer with respect to the amount of polymorphism exhibited is estimated as the polymorphic information content (PIC) value [57]. The allelic diversity parameters viz., major allele frequency, gene diversity and polymorphism information content for each marker loci were estimated using the PowerMarker version 3.25. The factorial analysis on dissimilarity of the accessions was carried out using DARwin v 6.0.14. The dissimilarity matrix prepared based on the presence or absence of a particular allele in a particular accession was used as the input for the metric multidimensional scaling or principal coordinate analysis (PCoA). The phylogenetic relationships among the accessions for LPA gene diversity were plotted as a dendrogram employing the un-weighted hierarchical clustering method. The associations between grain PA content and cgSSR markers, and the effect of marker on the phenotype were calculated following the standard method [58]. The BLUP values of PA content estimated combining two years data was used as phenotype input for association analysis. The association between PA and 14 novel candidate gene based SSR markers (cgSSR) used to scan the genome following mixed linear model (MLM) in GAPIT package [59] in R software. The marker *p* values calculated for each marker was considered to declare significant association; the marker with *p* ≤ 0.05 was declared as significantly associated with phenotype and phenotypic explained variation was estimated from the r^2^.

### Population parameters estimation for *SPDT* and *OsPT8* genes

In order to estimate the number of haplotypes, haplotype diversity (Hd), nucleotide diversity (Pi), and Tajima's D value, the *SPDT* and *OsPT8* gene sequences of 116 rice genotypes were retrieved from the database for Rice Functional Genomics and Breeding (https://www.rmbreeding.cn/Index/). The data was analysed with DnaSP (DNA Sequence Polymorphism) software package.

## Supplementary Information

**Additional file 1: Supplementary Fig. S1.** The grain PA content of 94 rice genotypes and two landraces for the year 2020 and 2021. **Supplementary Fig. S2.** Representative image of allelic diversity at the (AAG)n genetic loci of 3’ UTR of the SPDT gene among 96 rice genotypes (uncropped full length gel image). **Supplementary Fig. S3.** The dendrogram constructed based on the allelic diversity for PA-related candidate genes in 96 rice genotypes. **Supplementary Table S1.** Candidate genes reported to be associated with grain PA content in rice. **Supplementary Table S2.** Nucleotide diversity parameters for the SPDT and OsPT8 genes in rice.

## Data Availability

The phenotypic data on phytic acid content of rice genotypes analysed during the current study are available from the corresponding author on reasonable request. With regard to the gene sequence data, the datasets generated and/or analysed during the current study are publicly available in the Rice Genome Annotation Project (http://rice.uga.edu) in the following web links: (http://rice.uga.edu/cgi-bin/sequence_display.cgi?orf=LOC_Os02g57400; http://rice.uga.edu/cgi-bin/sequence_display.cgi?orf=LOC_Os03g52760; http://rice.uga.edu/cgi-bin/sequence_display.cgi?orf=LOC_Os04g55800; http://rice.uga.edu/cgi-bin/sequence_display.cgi?orf=LOC_Os04g56580; http://rice.uga.edu/cgi-bin/sequence_display.cgi?orf=LOC_Os03g04920; http://rice.uga.edu/cgi-bin/sequence_display.cgi?orf=LOC_Os06g05160; http://rice.uga.edu/cgi-bin/sequence_display.cgi?orf=LOC_Os10g30790)

## Abbreviations

cgSSR: Candidate gene-based simple sequence repeats
PA: Phytic acid
*SPDT*: SULTR-like phosphorus distribution transporter
