# Supplementary material for: Understanding natural genetic variation for grain phytic acid content and functional marker development for phytic acid-related genes in rice
Source: BMC Plant Biol. 2022 Sep 17;22:446. doi: 10.1186/s12870-022-03831-2 (PMC9482188; doi:10.1186/s12870-022-03831-2)
Supplement: Supplementary file 1 — Additional file 1: Supplementary Fig. S1. The grain PA content of 94 rice genotypes and two landraces for the year 2020 and 2021. Supplementary Fig. S2. Representative image of allelic diversity at the (AAG)n genetic loci of 3’ UTR of the SPDT gene among 96 rice genotypes (uncropped full length gel image). Supplementary Fig. S3. The dendrogram constructed based on the allelic diversity for PA-related candidate genes in 96 rice genotypes. Supplementary Table S1. Candidate genes reported to be associated with grain PA content in rice. Supplementary Table S2. Nucleotide diversity parameters for the SPDT and OsPT8 genes in rice. [file 12870_2022_3831_MOESM1_ESM.pdf]

Understanding natural genetic variation for grain phytic acid content and functional marker development for phytic acid-related genes in rice

Muhammed Azharudheen TP[1], Awadhesh Kumar[2], Anilkumar C[1], Rameswar Prasad Sah[1*], Sasmita Behera[1] and BC Marndi[1]

([1]Crop Improvement Division, ICAR-National Rice Research Institute, Cuttack, India; [2] Crop Physiology and Biochemistry Division, ICAR-National Rice Research Institute, Cuttack, India)

Supplementary Information

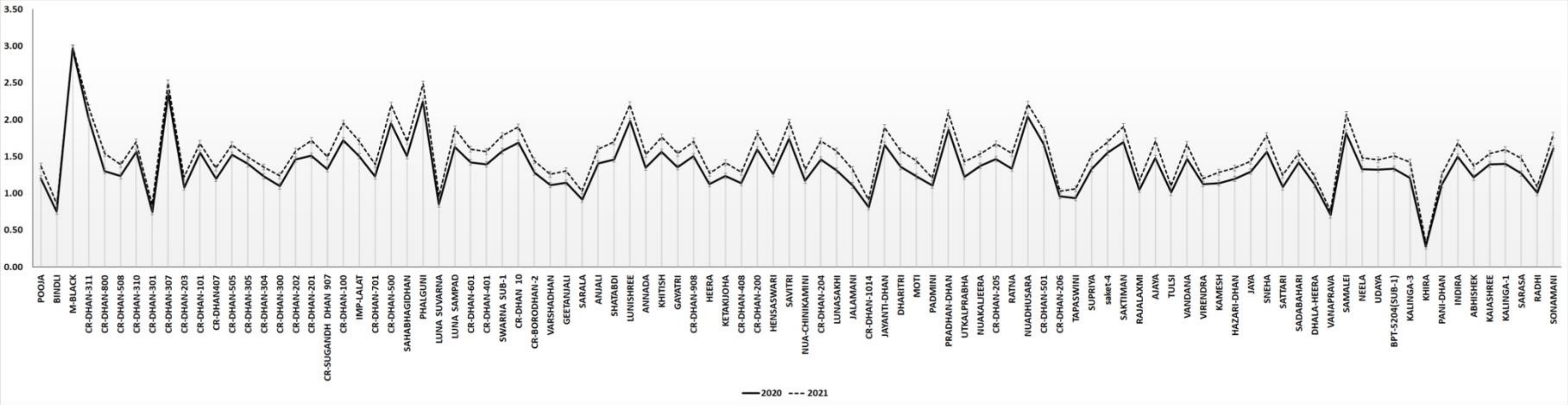

Supplementary Fig. S1. The grain PA content of 94 rice genotypes and two landraces for the year 2020 and 2021

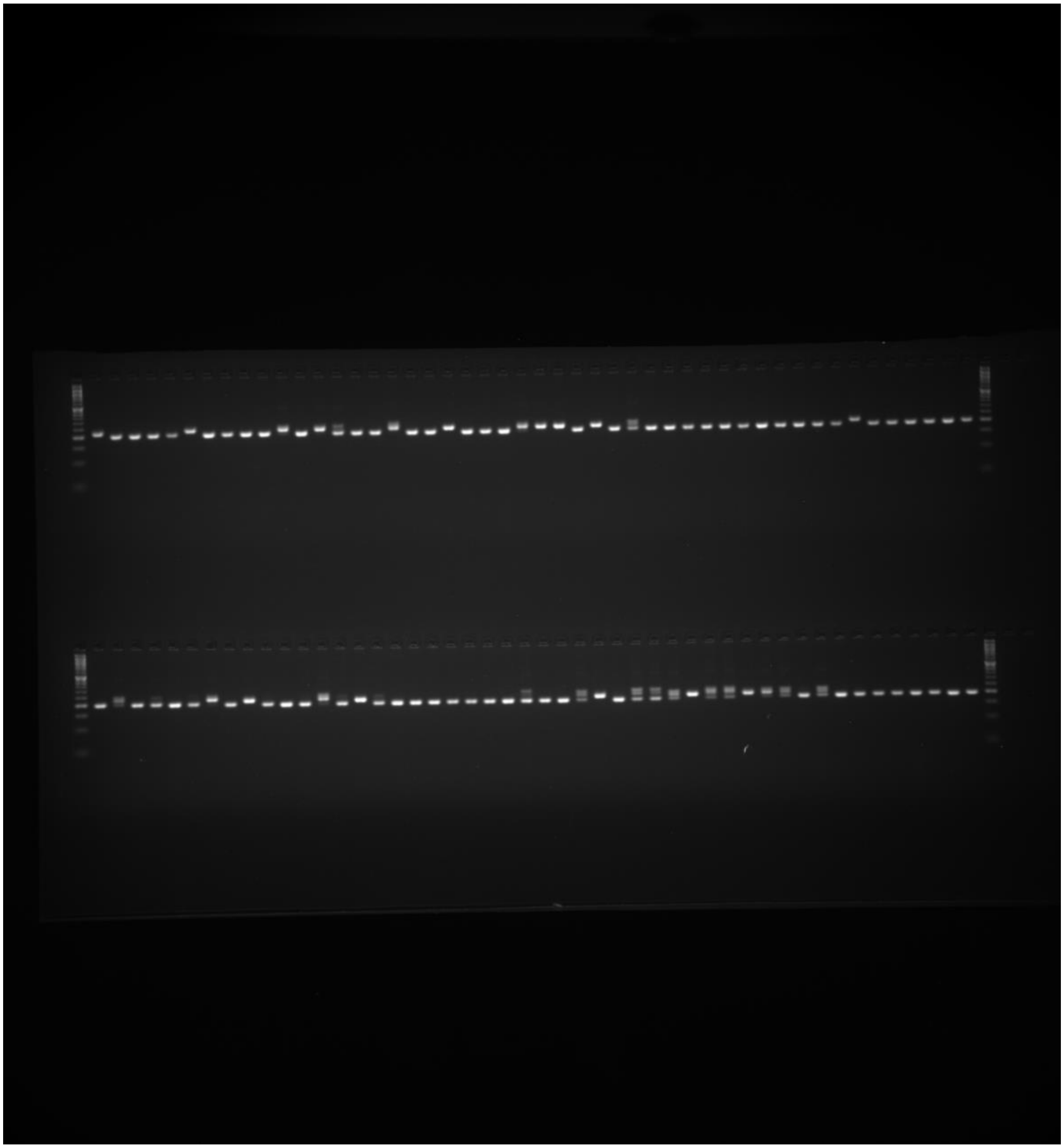

Supplementary Fig. S2. Representative image of allelic diversity at the (AAG)n genetic loci of 3’ UTR of the *SPDT* gene among 96 rice genotypes (uncropped full length gel image)

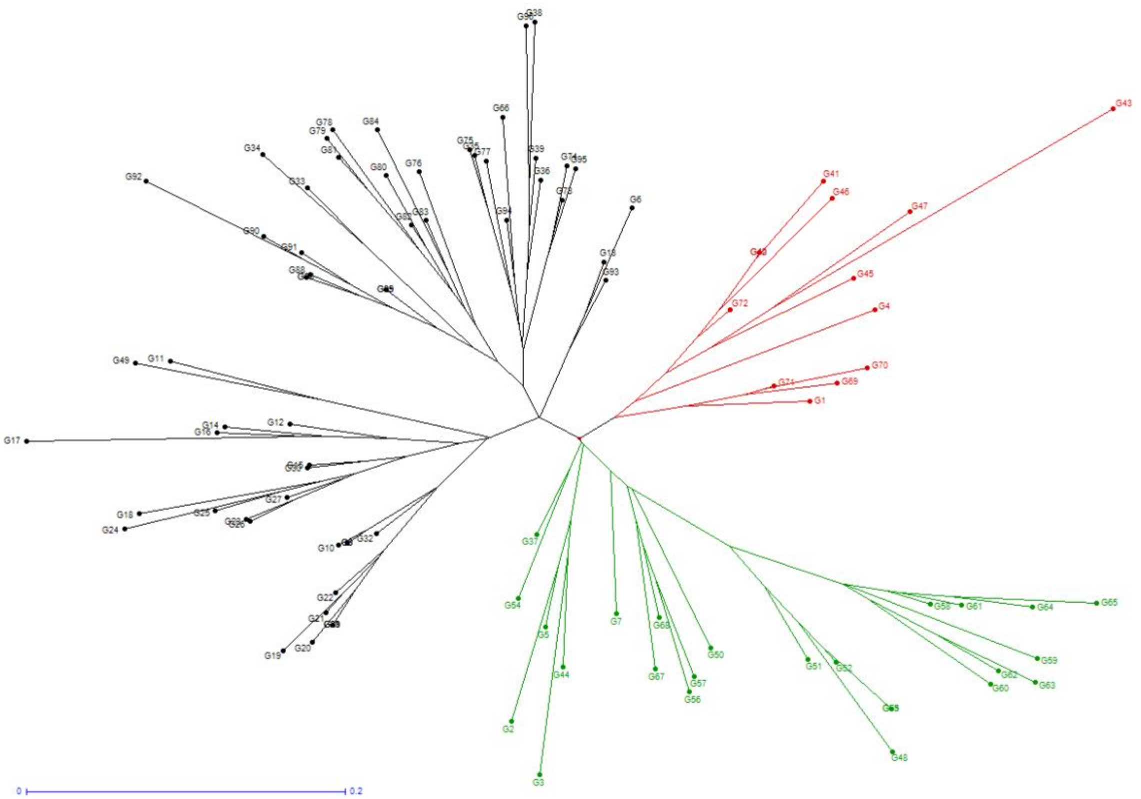

Supplementary Fig. S3. The dendrogram constructed based on the allelic diversity for PA-related candidate genes in 96 rice genotypes

Supplementary Table S1. Candidate genes reported to be associated with grain PA content in rice

Gene	Locus ID	Chr.	Gene size (bp)	Protein product	Putative function	Traits governed	Reference
OsLpa1	LOC_Os02g57400	2	1488	Kinase	Phytic acid biosynthetic pathway	Grain PA content	[1]
OsMRP5/ OsABCC13	LOC_Os03g04920	3	4518	Transporter	Putative multidrug resistance-associated protein involved in PA metabolism	Grain PA content	[2]
OsMIK	LOC_Os03g52760	3	1194	Kinase	Phytic acid biosynthetic pathway	Grain PA content	[1,3]
LPA	LOC_Os04g55800	4	1986	Transporter	Putative sulfate transporter, controls the grain metabolite profile.	Grain PA content	[4]
OsIPK1	LOC_Os04g56580	4	1338	Kinase	Catalyzes the last step of phytic acid biosynthesis in rice (phosphorylation of InsP5 molecule at the 2nd position)	Grain PA content	[5]
SPDT/ OsSultr3;4	LOC_Os06g05160	6	2013	Transporter	Controls the allocation of phosphorus to the grain	Grain P /PA content	[6-7]
OsPT8	LOC_Os10g30790	10	1626	Transporter	Phosphorus redistribution from source to sink organs and allocation between embryo and endosperm	Grain P content	[8]

Supplementary Table S2. Nucleotide diversity parameters for the SPDT and OsPT8 genes in rice

**Parameters**	**Gene**	
	**SPDT**	**OsPT8**
Number of sequences used	116	116
Number of sites/sequence length (bp)	6178	2821
Number of haplotypes (h)	95	115
Haplotype diversity (Hd)	0.988	0.9999
Variance of haplotype diversity	0.00003	0.0000013
Nucleotide diversity (Pi)	0.63237	0.68848
Theta (per site) from Eta	0.55262	0.56143
Average number of nucleotide differences (k)	3906.77841	1942.191
Tajima's D	0.48702 (P>0.10)	0.76348 (P>0.10)

References

1. Kim SI, Andaya CB, Newman JW, Goyal SS, Tai TH. Isolation and characterization of a low phytic acid rice mutant reveals a mutation in the rice orthologue of maize MIK. Theor. Appl. Genet. 2008;117(8):1291-301.
2. Tan Y, Zhou C, Goßner S, Li Y, Engel KH, Shu Q. Phytic acid contents and metabolite profiles of progenies from crossing low phytic acid OsMIK and OsMRP5 RICE (Oryza sativa L.) mutants. J. Agric. Food Chem. 2019;67(42):11805-14.
3. Zhao HJ, Cui HR, Xu XH, Tan YY, Fu JJ, Liu GZ, Poirier Y, Shu QY. Characterization of OsMIK in a rice mutant with reduced phytate content reveals an insertion of a rearranged retrotransposon. Theor. Appl. Genet. 2013;126(12):3009-20.
4. Zhou C, Tan Y, Goßner S, Li Y, Shu Q, Engel KH. Stability of the metabolite signature resulting from the OsSULTR3; 3 mutations in low phytic acid rice (Oryza sativa L.) seeds upon cross-breeding. J. Agric. Food Chem. 2018 Aug 15;66(35):9366-76.
5. Ali N, Paul S, Gayen D, Sarkar SN, Datta K, Datta SK. Development of low phytate rice by RNAi mediated seed-specific silencing of inositol 1, 3, 4, 5, 6-pentakisphosphate 2-kinase gene (IPK1). PloS one. 2013;8(7): e68161.
6. Yamaji N, Yuma T, Takaaki M, Namiki M, Kaoru TY, Jian Feng Ma. Reducing phosphorus accumulation in rice grains with an impaired transporter in the node. *Nature* 2017; 541(7635): 92-95.
7. Kumar A, Nayak S, Ngangkham U, Sah RP, Lal MK, Azharudheen TP, Behera S, Swain P, Behera L, Sharma S. A single nucleotide substitution in the SPDT transporter gene reduced phytic acid and increased mineral bioavailability from Rice grain (*Oryza sativa* L.). J. Food Biochem. 2021;45(7): e13822.

8. Li Y, Zhang J, Zhang X, Fan H, Gu M, Qu H, Xu G. Phosphate transporter OsPht1; 8 in rice plays an important role in phosphorus redistribution from source to sink organs and allocation between embryo and endosperm of seeds. Plant Sci 2015 Jan 1; 230:23-32.
